# Supplementary material for: Identification of the growth cone as a probe and driver of neuronal migration in the injured brain
Source: Nat Commun. 2024 Mar 9;15:1877. doi: 10.1038/s41467-024-45825-8 (PMC10924819; doi:10.1038/s41467-024-45825-8)
Supplement: Supplementary file 1 — Supplementary information [file 41467_2024_45825_MOESM1_ESM.pdf]

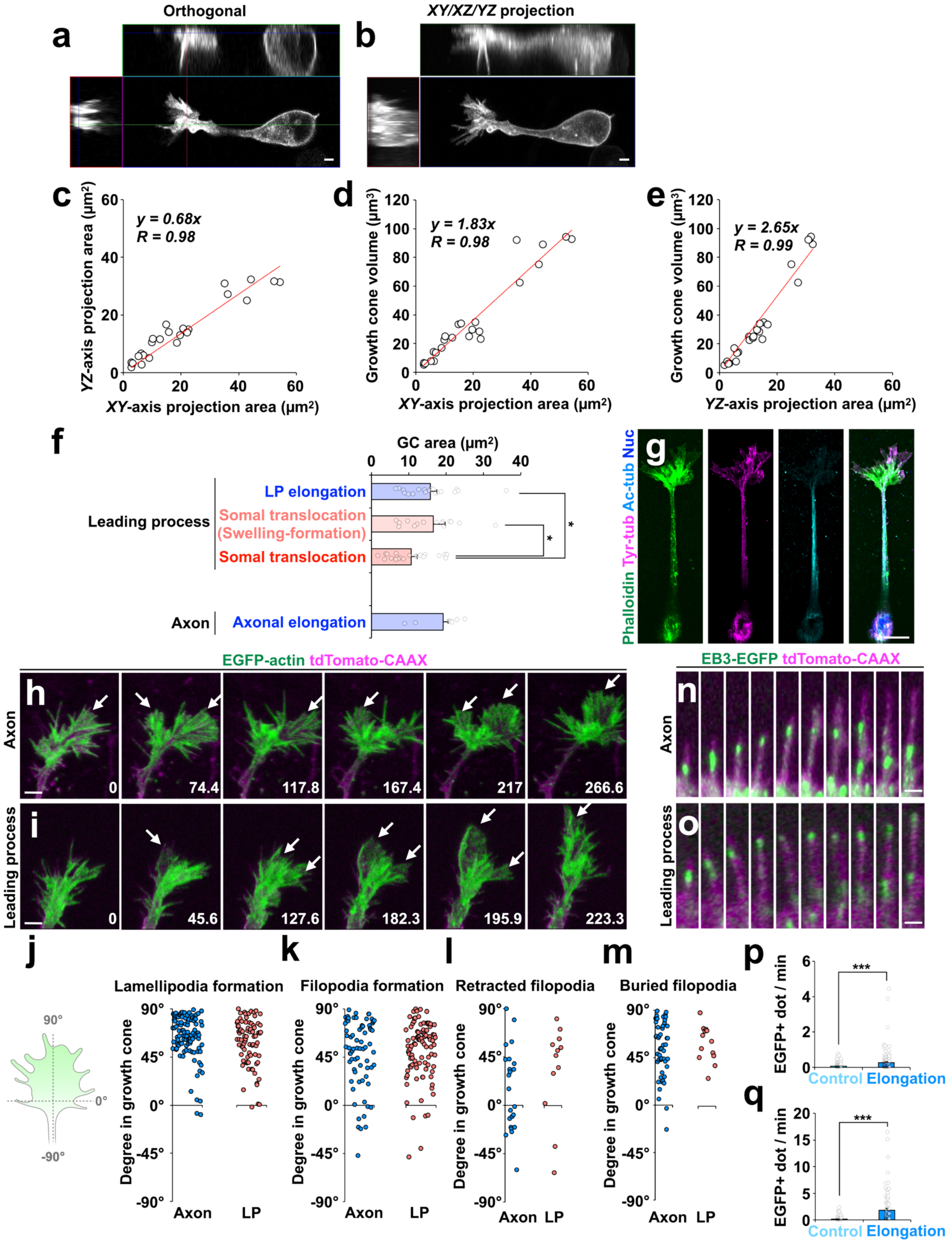

Supplementary Fig. 1: Commonality of morphology and dynamics in the growth cone of axons and leading process

(**a, b**) Orthogonal (a) and XY/XZ/YZ projection (b) images of Venus-CAAX-expressing cultured migrating neuron.

(**c-e**) Correlation between XY- and YZ projection area (c), XY-axis projection area and growth cone volume (d), and YZ-axis projection area and growth cone volume (e). R indicates Pearson's product-moment correlation coefficient.

(**f**) Growth cone area in the leading process of migrating neurons and axons of differentiating neurons.

(**g**) Representative images of an LP growth cone-like structure stained with phalloidin (green) and labeled with anti-tyrosinated-tubulin (magenta) and anti-acetylated-tubulin (cyan) antibodies shown in Fig. 1e. Nucleus is stained with Hoechst 33342 (blue).

(**h, i**) Time-lapse images of an EGFP-actin-expressing axonal growth cone (h) and LP growth cone-like structure (i). Arrows indicate lamellipodia.

(**j-m**) Distribution of formed lamellipodia (j), formed filopodia (k), retracted filopodia (l), and buried filopodia (m) in axonal growth cone (axon, blue) and LP growth cone-like structure (LP, red). Degree is defined in (j).

(**n-q**) Time-lapse images (n, o) and density (p, q) of an EB3-EGFP+ dots in a tdTomato-CAAX-labeled filopodium in axonal growth cone (n, p) and LP growth cone-like structure (o, q). Ten sequential images are shown.

Scale bars: a, b, h, i, 2 µm; g, 10 µm; n, o, 1 µm. **p* < 0.05, ****p* < 0.005. Error bars indicate mean ± SEM. For more detail, see the Source Data file.

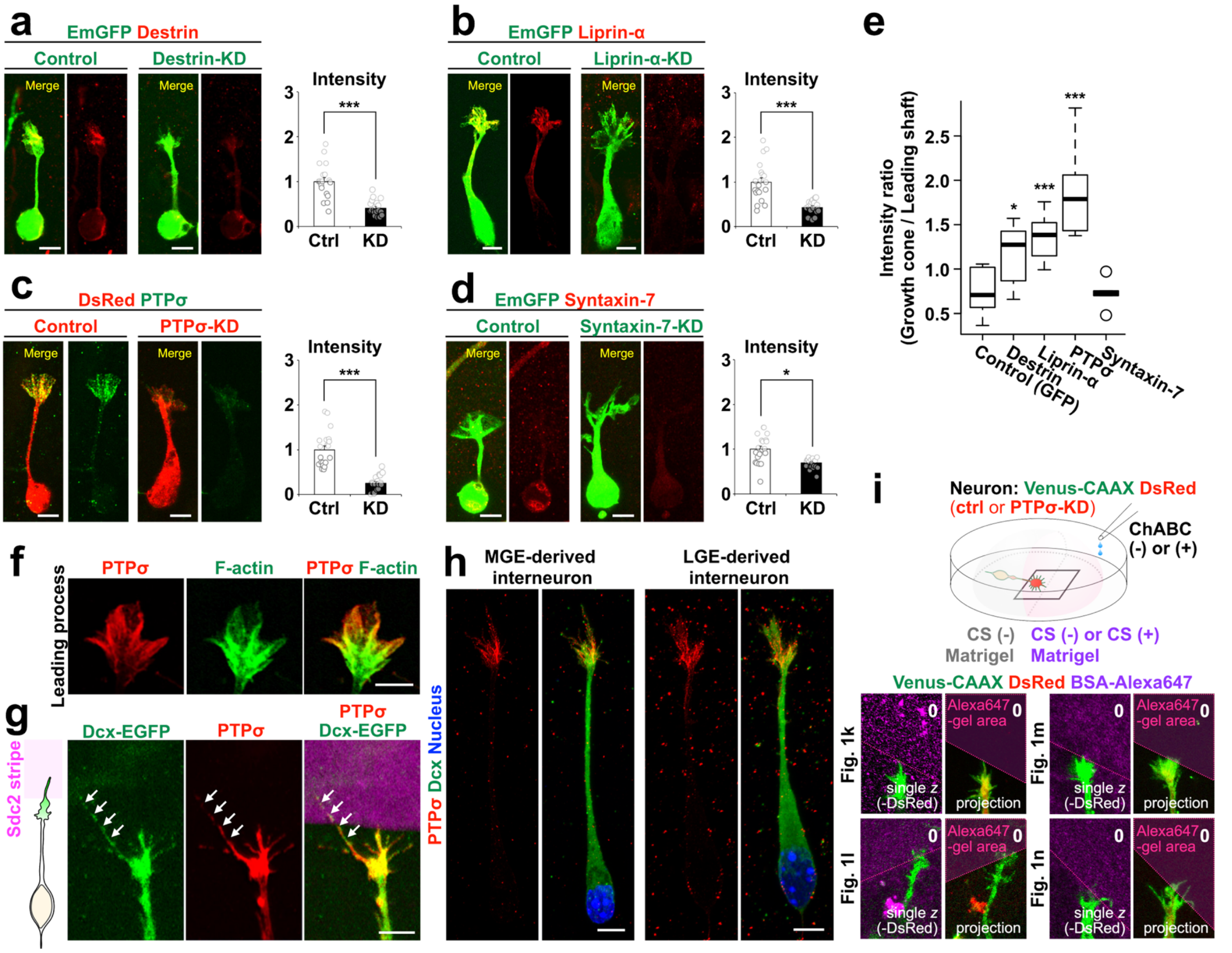

Supplementary Fig. 2: PTPσ is concentrated in the growth cone of migrating neurons

(**a-d**) Validation of anti-Destrin (a), Liprin-α (b), PTPσ (c), and Syntaxin-7 (d) antibodies. Representative images of EmGFP+ (a, b, d, green) or DsRed+ (c, red) neurons in which Destrin (a, red), Liprin-α (b, red), PTPσ (c, green), and Syntaxin-7 (d, red) were labeled. Expression levels of endogenous proteins were decreased by KD (graphs).

(**e**) Intensity ratio (protein concentration in growth cone) of Destrin, Liprin-α, PTPσ, and Syntaxin-7. Cytosolic EGFP expression served as a control. Data are expressed as box and whisker plots with individual data points (in detail: median, upper box bound (75%), lower box bound (25%), minus whisker, plus whisker).

(**f**) Colocalization of PTPσ (red) and F-actin (green) in an LP growth cone.

(**g**) Representative image of a leading filopodium (arrows) of a Dcx-EGFP+ neuron with GFP (green) and PTPσ (red). Magenta indicates Sdc2 stripe.

(**h**) Representative super-resolution images of an LP growth cone-like structure of MGE- and LGE-derived migrating interneurons expressing Dcx (green) and PTPσ (red). The nucleus is stained with Hoechst 33342 (blue).

(**i**) Original single z-plane images (left) and projection images (right) showing the border of Alexa647-containing (magenta) and non-colored Matrigels in Fig. 1k-n.

Scale bars, 5 μm. **p* < 0.05, ****p* < 0.005 (e, vs GFP). Error bars (a-d) indicate mean ± SEM. For more detail, see the Source Data file.

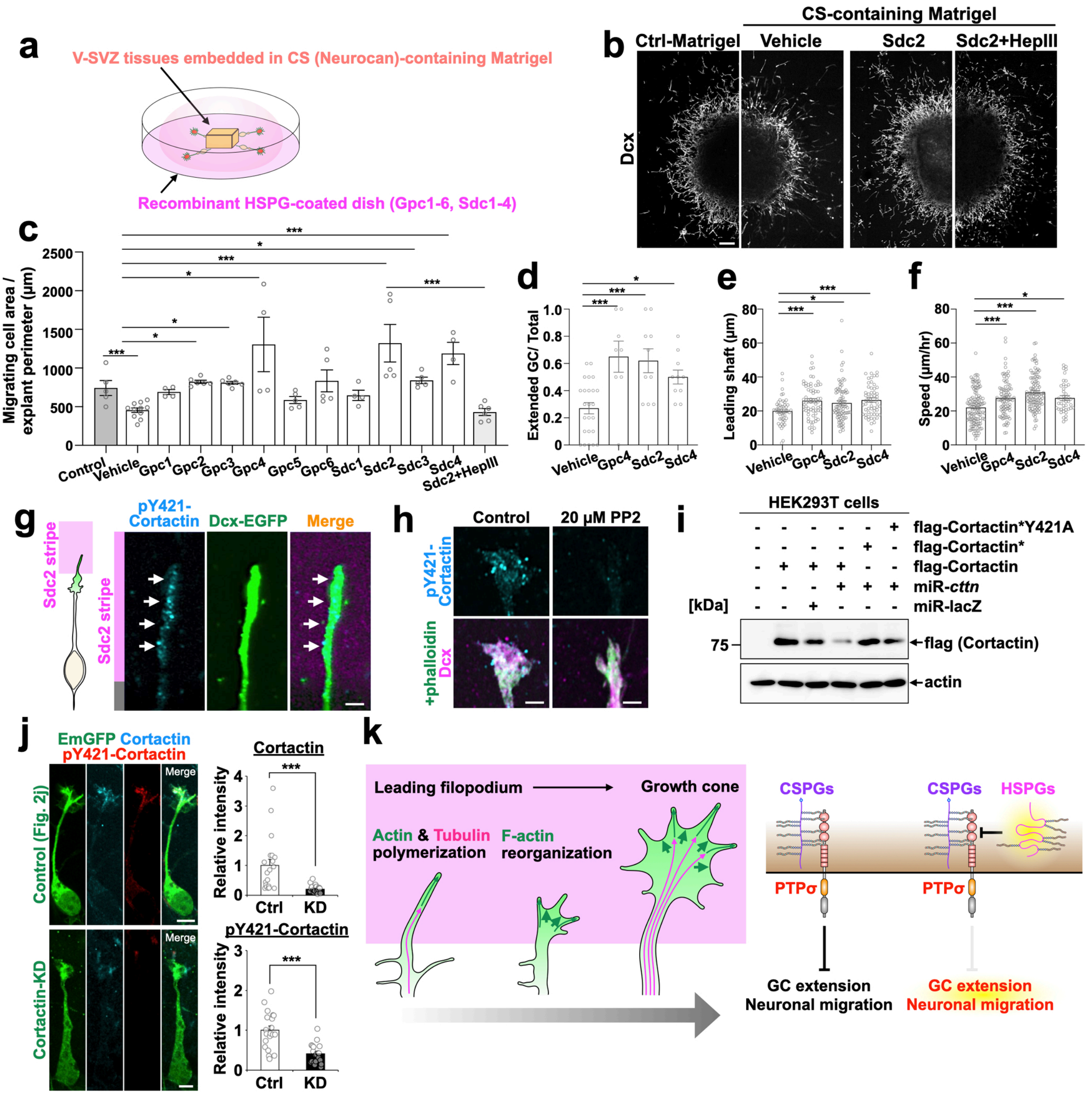

Supplementary Fig. 3: HSPGs promote neuronal migration in CS-containing Matrigel

(**a**) Experimental scheme.

(**b**) Representative images of Dcx-labelled migrating neurons cultured on dishes coated with Vehicle, Sdc2, and Sdc2 treated with Heparinase III in Matrigel with CS or without CS (Ctrl-Matrigel).

(**c**-**f**) The distribution area of migrating neurons (c), ratio of expanded growth cones (d), length of leading shafts (e), and migration speed (f) were determined by coating the dish bottom with recombinant HSPGs. Control cells were cultured in Matrigel, while the other samples were cultured in Matrigel containing CS.

(**g**) Representative image of a leading filopodium of Dcx-EGFP+ neuron stained for GFP (green) and pY421-Cortactin (cyan, arrows). Magenta indicates Sdc2 stripe.

(**h**) Localization of pY421-Cortactin (cyan) in the growth cones of control and PP2-treated Dcx+ (magenta) neurons. F-actin is stained with phalloidin.

(**i**) Western blot confirmation of Cortactin-KD and KD-resistant Cortactin expression.

(**j**) Validation of anti-Cortactin and pY421-Cortactin antibodies. Representative images of EmGFP+ (green) neurons labeled for Cortactin (cyan) and py421-cortactin (red) are shown. Enlarged images of a control cell were also shown in Fig. 2j. Expression levels of Cortactin and py421-cortactin are significantly decreased by KD.

(**k**) Model (left) and molecular mechanism (right) of leading filopodium and growth cone formation.

Scale bars: g, h, 2 μm; j, 5 μm; b, 100 μm. *$p < 0.05$, **$p < 0.01$, ***$p < 0.005$. Error bars indicate mean ± SEM. For more detail, see the Source Data file.

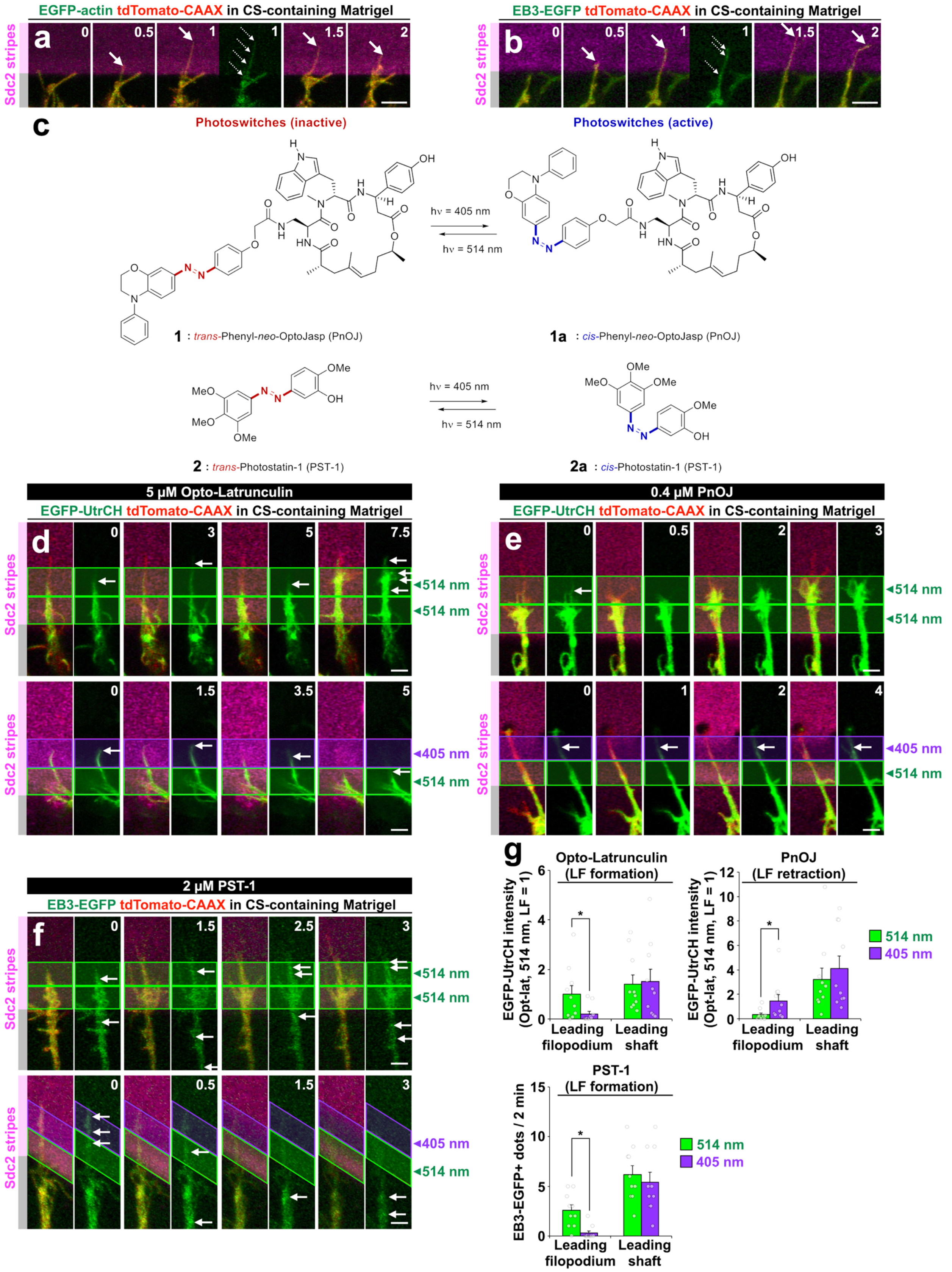

Supplementary Fig. 4: Structures and validation of photoswitchable inhibitors

(**a, b**) Time-lapse images of EGFP-actin (a, green) or EB3-EGFP (b, green)- and tdTomato-CAAX (red)-expressing migrating neurons on Sdc2 stripes (magenta) cultured in the CS-containing Matrigel. Dotted arrows indicate EGFP-actin (a) or EB3-EGFP+ dots (b) in the leading filopodium.

(**c**) Chemical structures of photoswitchable inhibitors. Inactive (**1**, **2**) and active configurations (**1a**, **2a**) of the chemicals are shown. Note that in this study Phenyl-*neo*-OptoJasp (PnOJ) was used (**1**, compound **13** in the reference[1]), which features a 10x longer deactivation half-life (t1/2 = 22 s) compared to *neo*-OptoJasp (nOJ; compound **6** in the reference[1], t1/2 = 2s).

(**d-f**) Time-lapse images of EGFP-UtrCH- (d, e) or EB3-EGFP- (f) (green) and tdTomato-CAAX (red)-expressing neurons on Sdc2 stripes (magenta) cultured with Opto-latrunculin (d), PnOJ (e), and PST-1 (f). Regions of illumination with 514 and 405 nm lasers are shown in green and purple boxes, respectively. Arrows indicate F-actin (d, e) and microtubule plus-end (f), respectively.

(**g**) Inhibitory effects of Opto-latrunculin, PnOJ, and PST-1 at the leading filopodium and leading shaft of migrating neurons.

Scale bars: a, b, 5 μm; d-f, 2 μm. **p* < 0.05. Error bars indicate mean ± SEM. For more detail, see the Source Data file.

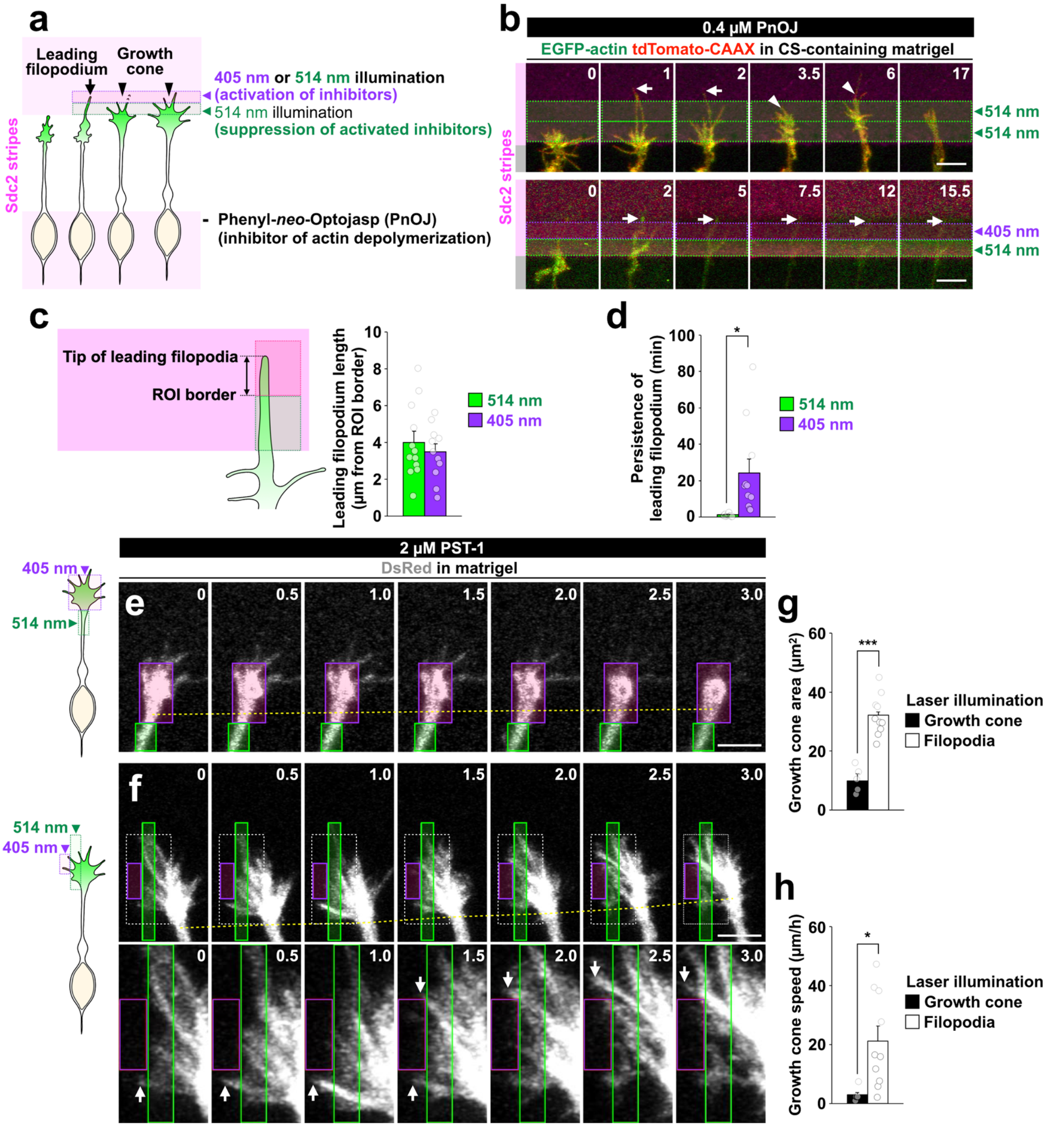

Supplementary Fig. 5: Manipulation of filopodia dynamics in the LP growth cones using photoswitchable inhibitors

(**a**) Experimental design using PnOJ.

(**b**) Time-lapse images of EGFP-actin (green)- and tdTomato-CAAX (red)-expressing migrating neurons on Sdc2 stripes (magenta) cultured with 0.4 μM PnOJ. Regions of illumination with 514 and 405 nm lasers are shown in green and purple boxes, respectively. Arrows and arrowheads indicate leading filopodium and growth cone, respectively.

(**c, d**) Length (c) and persistence (d) of leading filopodium following 405 nm-laser illumination.

(**e, f**) Time-lapse images of DsRed-expressing migrating neurons cultured with 2 μM Photostatin-1 (PST-1). Purple and green boxed areas are illuminated with 405 nm and 514 nm lasers, respectively. Yellow dotted lines indicate the root of growth cone (GC). In GC illumination experiment (e), PST-1 activated by 405 nm laser-illumination inhibited GC extension and movement. In filopodia illumination experiment (f), PST-1 activated by 405 nm laser-illumination inhibited elongation of filopodia within 405 nm-illuminated region, but not outside of the region (white arrows).

(**g, h**) Area (g) and speed (h) of growth cone following laser illumination of whole growth cone and filopodia.

Scale bars: b, e, f, 5 μm. **p* < 0.05, ****p* < 0.005. Error bars indicate mean ± SEM. For more detail, see the Source Data file.

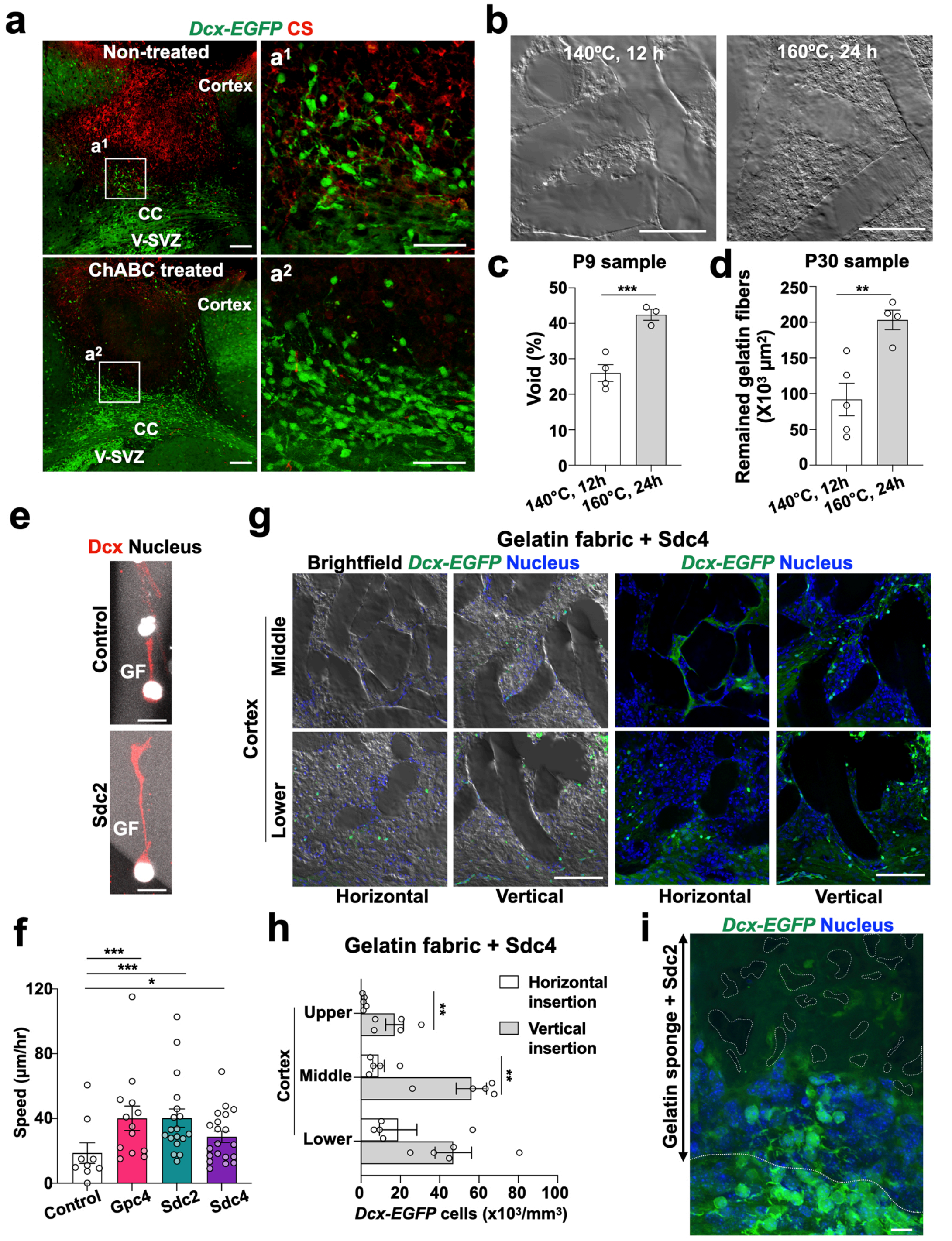

Supplementary Fig. 6: The neuronal migration in injured cortex with gelatin fabrics

(**a**) Representative images of CS expression in the P9 *Dcx-EGFP* mouse injured cortex. The brain sections were stained for GFP to detect Dcx-EGFP+ neurons (green) and CS (red). The brain section treated with ChABC prior to the primary antibody incubation showed no CS signals (lower panel), suggesting the CS signal in the non-treated cortical slice is not the background. Boxed areas are enlarged (a^1 and a^2).

(**b**) Brightfield image of P9 cortex with implanted GF. The fabrics manufactured by two different thermal cross-linking conditions were implanted into the P5 injured cortex. Brightfield images of gelatin fibers and surrounding tissues are shown.

(**c**) The percentage of void among gelatin fibers in the P9 injured cortex. The total area occupied by inserted fabric was counted as 100 %. A dot represents an analyzed brain.

(**d**) The area of remaining implanted gelatin fibers in the P30 brain. A dot represents an analyzed brain.

(**e**) Representative Z-stack confocal images of migrating neurons on the gelatin fibers. Tips of the Dcx (red) cells are on the gelatin fibers (white; autofluorescence). Nucleus is stained with Hoechst 33342 (white). Refer to Fig. 5f.

(**f**) Speed of cultured neurons on gelatin fibers containing Gpc4, Sdc2, or Sdc4 or gelatin fiber immersed in PBS (Control).

(**g**) Representative images of Sdc4-containing gelatin fibers and Dcx-EGFP+ neurons in P9 injured cortex. Brightfield images are overlaid with confocal images of Dcx-EGFP+ neurons (green) and Hoechst 33342 (blue) fluorescent cells.

(**h**) Distribution of Dcx-EGFP+ neurons in P9 injured cortex implanted with Sdc4-containing gelatin-fabric. Two different aligned gelatin fabrics (horizontal and vertical) were implanted. The graph shows the density of Dcx-EGFP+ neurons in the treated cortex. A dot represents an examined brain.

(**i**) Image of P9 *Dcx-EGFP* mouse cortex with Sdc2-enriched gelatin sponge. Gelatin sponge was inserted in the area demarcated by double arrows. Cavities of the inserted gelatin sponge are surrounded by dotted lines. Gelatin sponge walls (green; autofluorescence) hindered further GFP+ neuron migration above the cortex.

Scale bars: a, b, g, 100 μm; a^1, a^2, 50 μm; e, i, 10 μm. * $p < 0.05$, ** $p < 0.01$, *** $p < 0.005$. Error bars indicate mean ± SEM. For more detail, see the Source Data file.

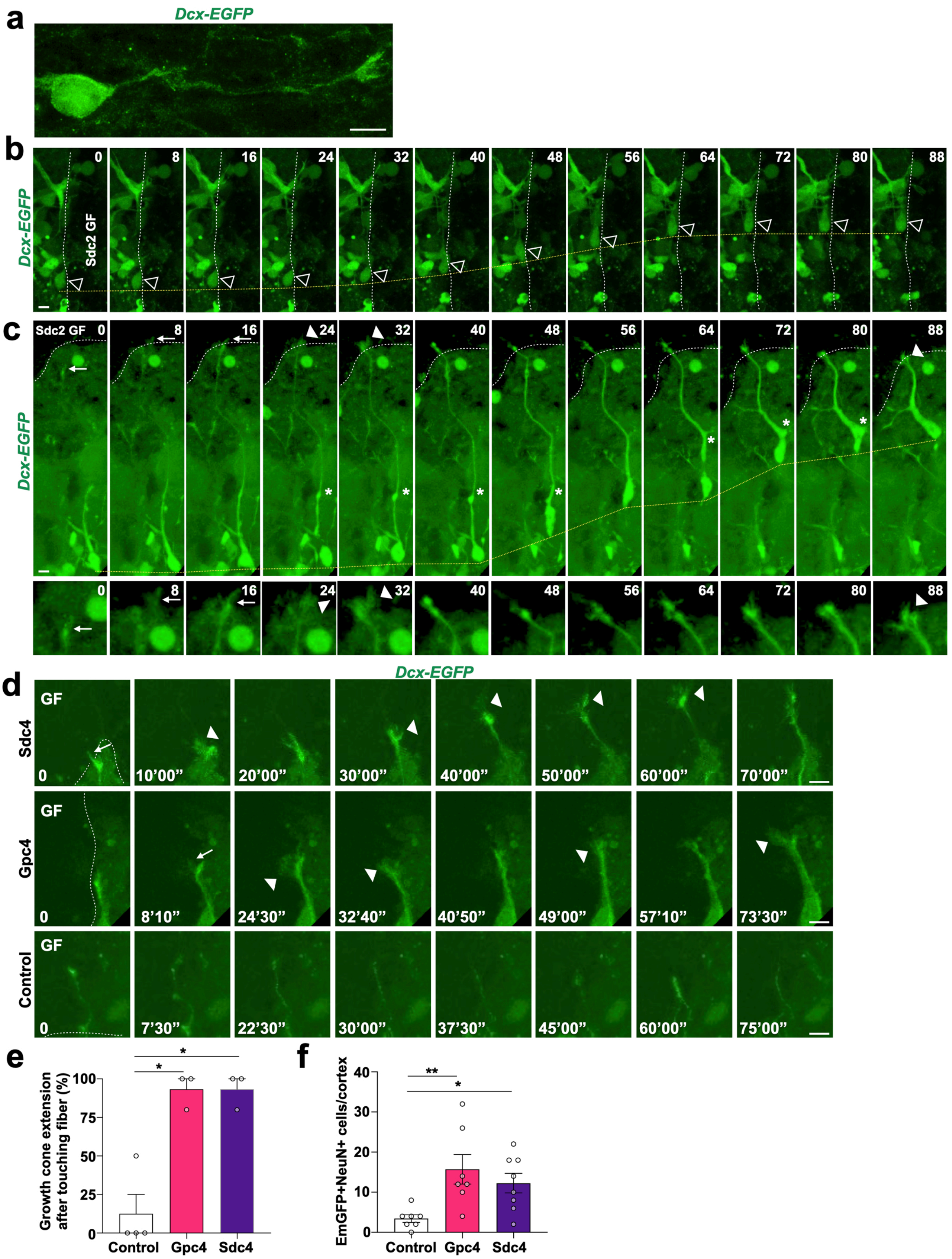

Supplementary Fig. 7: Gpc4 and Sdc4 promote growth cone extension and neuronal regeneration

(**a**) Representative super-resolution image of migrating neuron with extended growth cone at the tip of the leading process in injured cortex implanted with Sdc2-loaded gelatin fibers.

(**b, c**) Related to Fig. 6i and 6j. Time-lapse imaging of *Dcx-EGFP* brain slice cultures with Sdc2-containing gelatin fibers (the border of tissues and fibers are shown in white dot lines). Frames recorded every 8 min are shown. A Dcx-EGFP+ cell migrates along the fiber (b, open arrowheads) and another EGFP+ cell migrates a long distance toward the fiber (c). Yellow dotted lines show the position of the cell bodies. The growth cone of the EGFP+ cell (c) collapses and forms filopodia during the leading process elongation (arrows). The growth cone touches a gelatin fiber containing Sdc2 and extends its membrane (white arrowheads). The neuron forms swellings (asterisks) and continues somal translocations. Numbers indicate time in min.

(**d**) Time-lapse imaging of *Dcx-EGFP* brain slice culture with gelatin fibers (the border of tissues and fibers are shown in white dot lines). The collapsed growth cones elongate leading filipodia (arrows) and extend the membrane after touching gelatin fibers containing Gpc4 or Sdc4 (white arrowheads). While, the growth cone stays collapse after touching control gelatin fiber. Numbers indicate time in min and seconds.

(**e**) Dcx-EGFP+ cells of the brain slices acquired from the time-lapse imaging were quantitatively analyzed. The graph shows the percentage of growth cone extension after touching the gelatin fibers. A dot represents the imaged sample.

(**f**) Quantification of NeuN and EmGFP double-positive cells. The graph shows the number of EmGFP-labelled NeuN cells in P30 control, Gpc4- or Sdc4-containing gelatin fabric-implanted injured cortices.

Scale bars: a-d, 5 µm. **p* < 0.05, ***p* < 0.01. Error bars indicate mean ± SEM. For more detail, see the Source Data file.

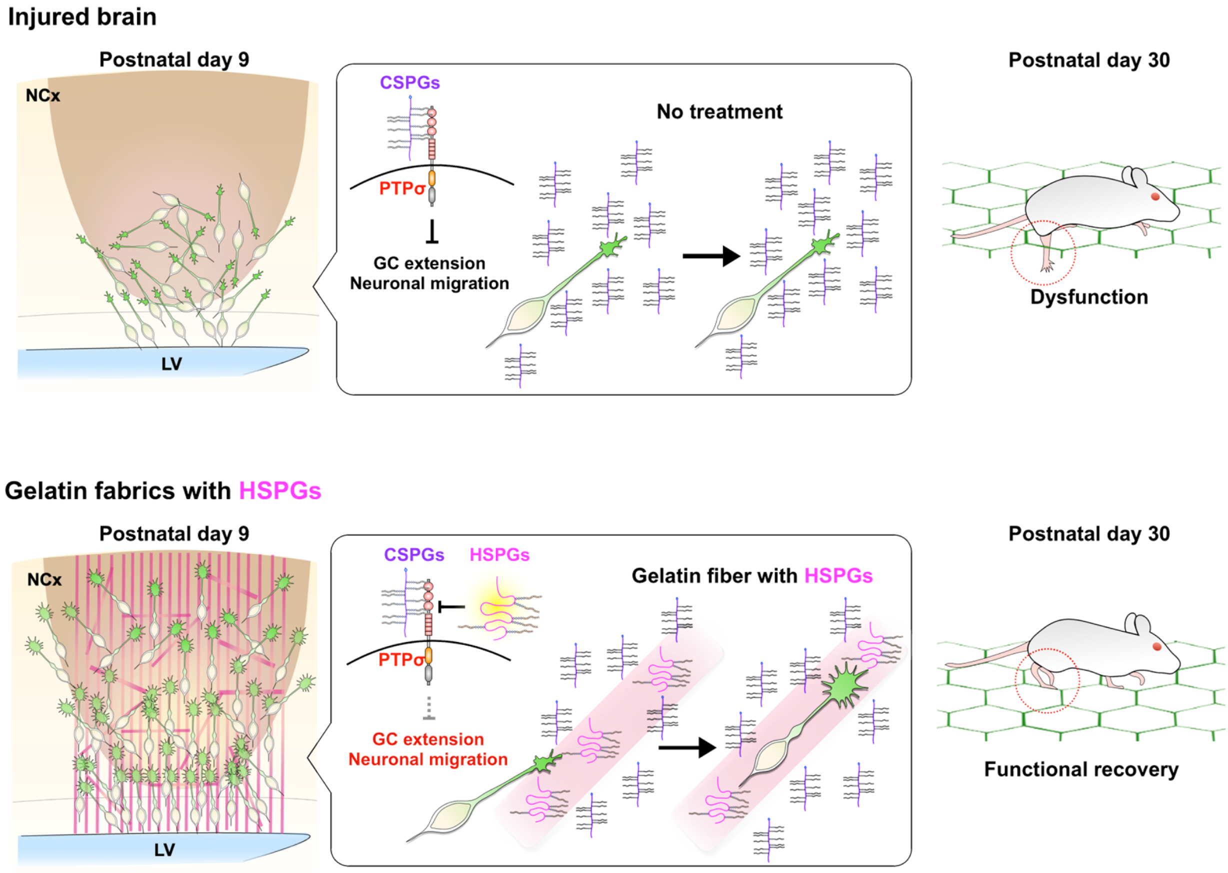

Supplementary Fig. 8: Schematic illustration of the summary of this study

Schematic illustration of the results of implantation of HSPGs-containing gelatin fabrics into CS-enriched injured cortices (Bottom) and unimplanted control (Top). The HSPGs-containing gelatin fabrics facilitate the extension of growth cones (GC), neuronal migration, and functional recovery after cortical injury. The middle panels show the regulatory mechanisms and function of membrane-bound PTPσ in GCs.

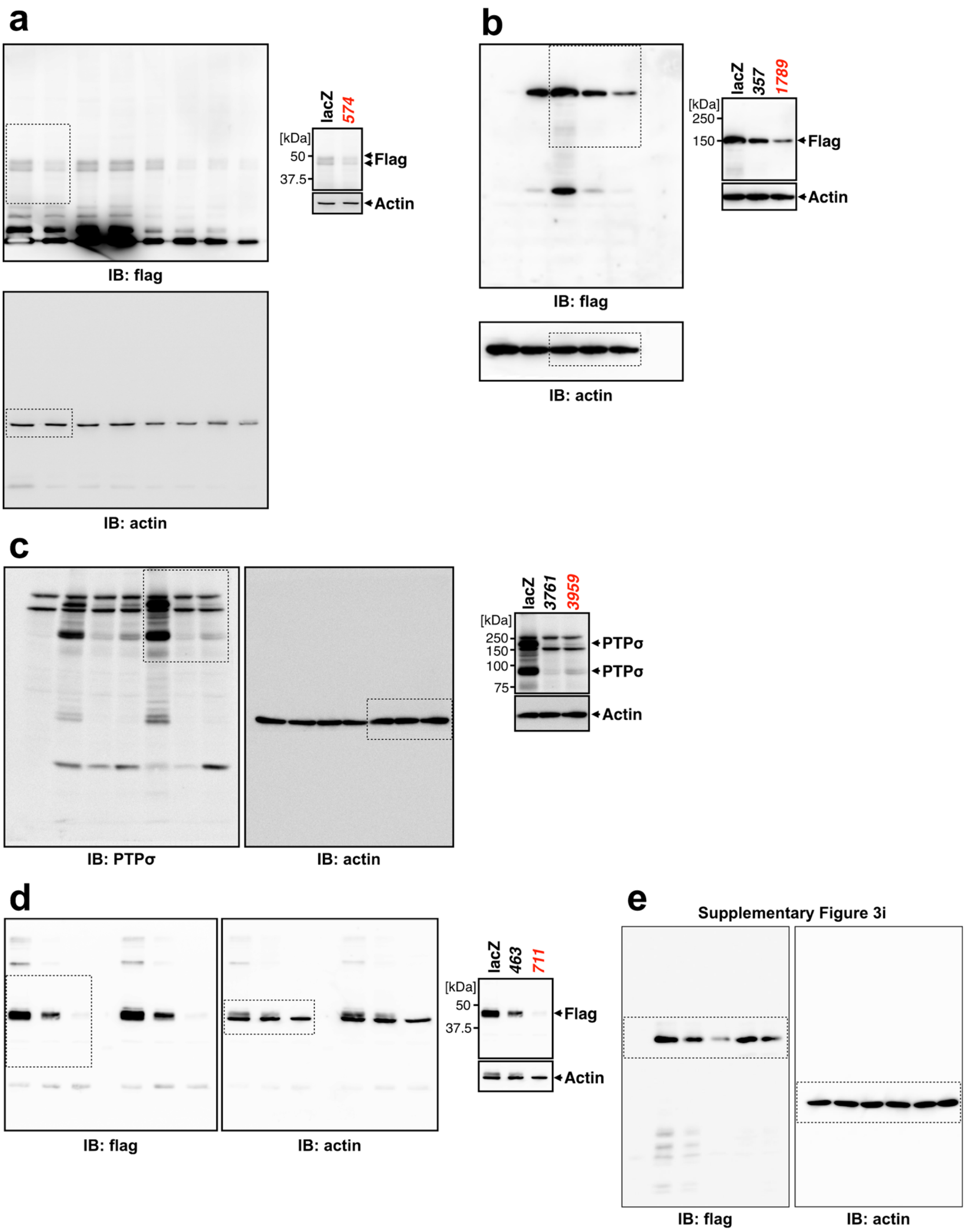

Supplementary Fig. 9: Western blot analyses

(**a**-**d**) Western blot images of knock-down experiments for Destrin (a), Liprin-α (b), PTPσ (c), and Syntaxin-7 (d). HEK-293T cells were transfected with plasmids and obtained cell lysates were subjected to the western blotting analyses. Dotted line areas are cropped and shown on the right. Numbers indicate the starting positions of designed miRNA oligos on target genes (cf. Supplementary Data1 for sequence). The miR oligos showing high efficiency of knockdown (red) were used in the other knockdown experiments (Supplementary Fig. 2a-d). The experiment was performed once targeting Destrin, Liprin-α, and Syntaxin-7. The experiment was repeated twice targeting PTPσ knock-down with similar results.

(**e**) Original western blot images of blots shown in Supplementary Fig. 3i. Dotted line areas are cropped and shown in Supplementary Fig. 3i. The experiment was repeated twice with similar results.

Reference

1. Küllmer, F. *et al.* Next Generation Opto-Jasplakinolides Enable Local Remodeling of Actin Networks. *Angew. Chemie Int. Ed.* (2022), 61, e202210220
