# Supplementary material for: Identification of the growth cone as a probe and driver of neuronal migration in the injured brain
Source: Nat Commun. 2024 Mar 9;15:1877. doi: 10.1038/s41467-024-45825-8 (PMC10924819; doi:10.1038/s41467-024-45825-8)
Supplement: Supplementary file 3 — Description of Additional Supplementary Files [file 41467_2024_45825_MOESM3_ESM.pdf]

Description of Additional Supplementary Files

Supplementary Data Legends:

File Name: Supplementary Data 1

Description: Data obtained from proteomic analyses

File Name: Supplementary Data 2

Description: Sequences of oligonucleotides.

Supplementary Movie Legends:

File Name: Supplementary Movie 1

Description: Time-lapse imaging of the axonal growth cone and the leading process (LP) growth cone-like structure The videos show Venus-CAAX-labeled axon and LP of differentiating and migrating neuron and an EGFP-actin (green)- and tdTomato-CAAX (magenta)-expressing axonal growth cone and LP growth cone-like structure.

File Name: Supplementary Movie 2

Description: Time-lapse imaging of Venus-CAAX (green)- and DsRed (red)-expressing migrating neurons on control or Sdc2 stripes (magenta) cultured in the CS-containing Matrigel

File Name: Supplementary Movie 3

Description: Time-lapse imaging of Venus-CAAX (green)- and DsRed (red)-expressing migrating neurons on Sdc2 stripes (magenta) cultured with 5 µM Opto-Latrunculin or 2 µM Photostatin-1 Regions of illumination with 514 and 405 nm laser are shown in green and purple boxes, respectively.

File Name: Supplementary Movie 4

Description: Three-dimensional reconstruction of the LP growth cones in the injured cortex File Name:

Supplementary Movie 5

Description: Time-lapse imaging of slice culture from P8 Dcx-EGFP cryoinjured cortex EGFP+ neurons migrate toward the injured site and turn back toward V-SVZ (Crop 1 and 2).

File Name: Supplementary Movie 6

Description: Gelatin fabrics consist of long aligned fibers with voids among the fibers Images show the alignment distribution of gelatin fibers and void in the gelatin structure.

File Name: Supplementary Movie 7

Description: Gelatin fibers are effective for promoting neuronal migration by adding Sdc2 V-SVZ-derived neurons cultured in CScontaining Matrigel migrate faster along the gelatin fiber with Sdc2 (Sdc2) than along the fiber without Sdc2 (Control).

File Name: Supplementary Movie 8

Description: Time-lapse imaging of the slice culture from P7 Dcx-EGFP cryoinjured cortex with implanted Sdc2-containing gelatin fabrics Two sequential movies show that 1) the neuron migrates (arrowheads point to the positions of cell body) along the gelatin fiber (white shaded area) and 2) the neuron migrates (arrowheads) from a distant position with pronounced process elongation, swelling formation and growth cone extension (asterisk).

File Name: Supplementary Movie 9

Description: Time-lapse imaging of the slice cultures from P7 Dcx-EGFP cryoinjured cortex with implanted Gpc4 or Sdc4-containing GFs Migrating neurons extend their growth cones after contacting gelatin fibers (white shaded area) enriched with Gpc4 or Sdc4; the growth cone keep collapsing after contacting control gelatin fibers (white shaded area).
