# Supplementary material for: Identification of the growth cone as a probe and driver of neuronal migration in the injured brain
Source: Nat Commun. 2024 Mar 9;15:1877. doi: 10.1038/s41467-024-45825-8 (PMC10924819; doi:10.1038/s41467-024-45825-8)
Supplement: Supplementary file 15 — Reporting Summary [file 41467_2024_45825_MOESM15_ESM.pdf]

nature portfolio

Corresponding author(s): Kazunobu Sawamoto

Last updated by author(s): Jan 19, 2024

# Reporting Summary

Nature Portfolio wishes to improve the reproducibility of the work that we publish. This form provides structure for consistency and transparency in reporting. For further information on Nature Portfolio policies, see our Editorial Policies and the Editorial Policy Checklist.

## Statistics

For all statistical analyses, confirm that the following items are present in the figure legend, table legend, main text, or Methods section.

| n/a | Confirmed | |
|---|---|---|
| ☐ | ☒ | The exact sample size (*n*) for each experimental group/condition, given as a discrete number and unit of measurement |
| ☐ | ☒ | A statement on whether measurements were taken from distinct samples or whether the same sample was measured repeatedly |
| ☐ | ☒ | The statistical test(s) used AND whether they are one- or two-sided<br>*Only common tests should be described solely by name; describe more complex techniques in the Methods section.* |
| ☐ | ☒ | A description of all covariates tested |
| ☐ | ☒ | A description of any assumptions or corrections, such as tests of normality and adjustment for multiple comparisons |
| ☐ | ☒ | A full description of the statistical parameters including central tendency (e.g. means) or other basic estimates (e.g. regression coefficient) AND variation (e.g. standard deviation) or associated estimates of uncertainty (e.g. confidence intervals) |
| ☒ | ☐ | For null hypothesis testing, the test statistic (e.g. *F*, *t*, *r*) with confidence intervals, effect sizes, degrees of freedom and *P* value noted<br>*Give P values as exact values whenever suitable.* |
| ☒ | ☐ | For Bayesian analysis, information on the choice of priors and Markov chain Monte Carlo settings |
| ☒ | ☐ | For hierarchical and complex designs, identification of the appropriate level for tests and full reporting of outcomes |
| ☐ | ☒ | Estimates of effect sizes (e.g. Cohen's *d*, Pearson's *r*), indicating how they were calculated |

*Our web collection on statistics for biologists contains articles on many of the points above.*

## Software and code

Policy information about availability of computer code

| | |
|---|---|
| Data collection | Zeiss ZEN 2.3 SP1 FP3 (64bit), Olympus FV3000 Fluoview FV31S-SW (ver. 2.6), and Keyence BZ-X800 Viewer/Analyzer (ver.1.1.1.8). |
| Data analysis | BellCurve for Excel (ver. 3.2), GraphPad Prism 10 (ver. 10.0.0) , Zeiss ZEN 3.6 (Blue edition), and Image J (ver. 1.53 or 1.54). |

For manuscripts utilizing custom algorithms or software that are central to the research but not yet described in published literature, software must be made available to editors and reviewers. We strongly encourage code deposition in a community repository (e.g. GitHub). See the Nature Portfolio guidelines for submitting code & software for further information.

## Data

Policy information about availability of data

All manuscripts must include a data availability statement. This statement should provide the following information, where applicable:

- Accession codes, unique identifiers, or web links for publicly available datasets
- A description of any restrictions on data availability
- For clinical datasets or third party data, please ensure that the statement adheres to our policy

Source data are provided with this paper. The mass spectrometry proteomics data generated in this study have been deposited to the ProteomeXchange Consortium via the PRIDE partner repository under accession code PXD048878. Additional data supporting the findings are provided in the Supplementary Information.

## Research involving human participants, their data, or biological material

Policy information about studies with human participants or human data. See also policy information about sex, gender (identity/presentation), and sexual orientation and race, ethnicity and racism.

| | |
|---|---|
| Reporting on sex and gender | N/A |
| Reporting on race, ethnicity, or other socially relevant groupings | N/A |
| Population characteristics | N/A |
| Recruitment | N/A |
| Ethics oversight | N/A |

Note that full information on the approval of the study protocol must also be provided in the manuscript.

# Field-specific reporting

Please select the one below that is the best fit for your research. If you are not sure, read the appropriate sections before making your selection.

[x] Life sciences  [ ] Behavioural & social sciences  [ ] Ecological, evolutionary & environmental sciences

For a reference copy of the document with all sections, see nature.com/documents/nr-reporting-summary-flat.pdf

# Life sciences study design

All studies must disclose on these points even when the disclosure is negative.

| | |
|---|---|
| Sample size | Sample sizes were chosen based on the prior studies experienced in the laboratory.<br>Ref. https://doi.org/10.1016/j.biomaterials.2023.122003, https://doi.org/10.15252/embj.201797404 |
| Data exclusions | No data was excluded from the analysis. |
| Replication | In vitro and in vivo experiments were replicated and data from at least three independent experiments were obtained. |
| Randomization | Animals were randomly assigned to all of the performed experiments. |
| Blinding | Experiments were not blinded, however, each experiment was paired with appropriate controls and samples were collected and analyzed under identical conditions. |

# Reporting for specific materials, systems and methods

We require information from authors about some types of materials, experimental systems and methods used in many studies. Here, indicate whether each material, system or method listed is relevant to your study. If you are not sure if a list item applies to your research, read the appropriate section before selecting a response.

### Materials & experimental systems

| n/a | Involved in the study | |
|---|---|---|
| [ ] | [x] | Antibodies |
| [ ] | [x] | Eukaryotic cell lines |
| [x] | [ ] | Palaeontology and archaeology |
| [ ] | [x] | Animals and other organisms |
| [x] | [ ] | Clinical data |
| [x] | [ ] | Dual use research of concern |
| [x] | [ ] | Plants |

### Methods

| n/a | Involved in the study | |
|---|---|---|
| [x] | [ ] | ChIP-seq |
| [x] | [ ] | Flow cytometry |
| [x] | [ ] | MRI-based neuroimaging |

## Antibodies

| | |
|---|---|
| Antibodies used | For IF<br>The following primary antibodies were used in the immunocytochemical screening of growth cone molecules (1:100 dilution for all antibodies):<br>rabbit anti-CaMKV (polyclonal, self-made), rabbit anti-LETM1 (polyclonal, self-made), rabbit anti-PICALM (polyclonal, self-made), |

| | |
|---|---|
| | rabbit anti-Fish (polyclonal, self-made), rabbit anti-Rab35 (polyclonal, self-made), rabbit anti-Arhgdia (polyclonal, self-made), rabbit anti-MARCKSL1 (polyclonal, self-made), rabbit anti-Tmod2 (polyclonal, self-made), rabbit anti-pGAP43 (S96) (polyclonal, self-made), rabbit anti-pRtn1 (polyclonal, self-made), rabbit anti-SCG10 (polyclonal, self-made), rabbit anti-pMAP1B (S25) (polyclonal, selfmade), mouse anti-Ap2a1 (610501, Becton Dickinson), mouse anti-Ap2b1 (610381, Becton Dickinson), rabbit anti-Arhgdia (sc-360, Santa Cruz), mouse anti-Calnexin (610523, Becton Dickinson), rabbit anti-Camk2 (MA1-047, Thermo Fisher Scientific), rabbit anti-Cofilin (C8736, Sigma-Aldrich), rabbit anti-Destrin (D8815, Sigma-Aldrich), mouse anti-PP2A (601555, Becton Dickinson), chicken anti-Liprinα (PPFIA1) (GW21470, Sigma-Aldrich), chicken anti-PTPσ (PTPRS) (GW21486, Sigma-Aldrich), mouse anti-Rtn4 (612238, Becton Dickinson), rabbit anti-Scamp (121002, Synaptic Systems), rabbit anti-Snap29 (111303, Synaptic Systems), rabbit anti-Stx7 (110073, Synaptic Systems), mouse anti-Stx8 (611352, Becton Dickinson), and mouse anti-Vcp (MA3-004, Thermo Fisher Scientific).<br><br>The following primary antibodies were used in the immunocytochemistry:<br>mouse monoclonal anti-acetylated tubulin (1:200, T6793, Sigma Aldrich), mouse monoclonal anti-cortactin (1:100, 05-180, Millipore); rabbit anti-pY421-cortactin (1:100, 4569S, Cell Signaling Technology), rabbit anti-pY421-Cortactin (1:100, AB3852, Sigma-Aldrich), rabbit anti-Destrin (1:100, D8815, Sigma Aldrich), rabbit anti-Stx7 (1:200, 110073, Synaptic Systems), rat anti-tyrosinated tubulin (1:200, MAB1864-I, Sigma Aldrich), rabbit anti-PPFIA1 (1:100, HPA042271, Sigma Aldrich), chicken anti-Liprinα (PPFIA1) (1:100, GW21470, Sigma-Aldrich), chicken anti-PTPσ (1:100, GW21486, Sigma Aldrich), rabbit anti-DsRed (1:1000, 632496, Clontech), rabbit anti-GFP (1:1000, 598, MBL life science), rat anti-GFP (1:1,000, 04404-84, Nacalai), and rabbit anti-DCX (1:500, 4604, Cell Signaling Technologies).<br><br>The following primary antibodies were used in the immunohistochemistry:<br>rabbit anti-DCX (1:500, 4604, Cell Signaling Technologies), mouse anti-CS56 (1:500, C-8035, Sigma Aldrich), chicken anti-PTPRS (1:200, GW21486, Sigma Aldrich), rat anti-GFP (1:500, 04404-84, Nacalai), and rabbit anti-NeuN (1:200, ab177487, Abcam).<br><br>For WB<br>rabbit anti-DYKDDDK antibody (1:1,000, 2368, Cell Signaling Technology), goat anti-PTPσ antibody (1:1,000, AF3430, R&D systems), and mouse monoclonal anti-actin antibody (1:10,000, MAB1501, Millipore). |
| Validation | For critical antibodies, control tissues and cells were used for self-validation. Cortactin, Destrin, Liprin-α, PTPσ, and Syntaxin-7 antibodies used in cultured neurons were validated by KD experiments. CS56 staining in injured brain sections was validated by digesting CS in sections with Chondroitinase ABC treatment. All the purchased antibodies used in the study were all validated for the application and species on the manufacturers' websites. The self-made antibodies used in the study were validated in the previous studies (DOIs; 10.1073/pnas.0904092106, 10.1186/s13041-021-00755-0, 10.1016/j.isci.2018.05.019, 10.1186/s13041-019-0510-z, and 10.1016/j.celrep.2023.113195.) |

## Eukaryotic cell lines

Policy information about cell lines and Sex and Gender in Research

| | |
|---|---|
| Cell line source(s) | HEK293T cells (from Dr. Yuki Hirota, Keio University) |
| Authentication | The cell line was not authenticated. |
| Mycoplasma contamination | The cell line was not tested for mycoplasma contamination. |
| Commonly misidentified lines (See ICLAC register) | No commonly misidentified cell lines were used in the study. |

## Animals and other research organisms

Policy information about studies involving animals; ARRIVE guidelines recommended for reporting animal research, and Sex and Gender in Research

| | |
|---|---|
| Laboratory animals | C57BL/6J (Japan SLC), Institute of Cancer Research (ICR) (Japan SLC), Dcx-EGFP (MMRRC, Cat#000244-MU; RRID: MMRRC_000244-MU) , and NSE-DTA mice (Dr. Sigetoshi Itohara) were used.  Detail information is provided in Methods section. Postnatal day 0 to 1 mice were used for in vitro and postnatal day 0 to 30 mice were used for in vivo. |
| Wild animals | No wild animals were used in this study. |
| Reporting on sex | Both sex were used in this study. |
| Field-collected samples | The study did not involve field-collected samples. |
| Ethics oversight | The animal experiments were performed in accordance with the guidelines and regulations of Nagoya City University. |

Note that full information on the approval of the study protocol must also be provided in the manuscript.

## Plants

| | |
|---|---|
| Seed stocks | N/A |
| Novel plant genotypes | N/A |
| Authentication | N/A |
